# High Levels of EBV-Encoded RNA 1 (EBER1) Trigger Interferon and Inflammation-Related Genes in Keratinocytes Expressing HPV16 E6/E7

**DOI:** 10.1371/journal.pone.0169290

**Published:** 2017-01-05

**Authors:** Sirinart Aromseree, Jaap M. Middeldorp, Chamsai Pientong, Monique van Eijndhoven, Octavia Ramayanti, Sinéad M. Lougheed, D. Michiel Pegtel, Renske D. M. Steenbergen, Tipaya Ekalaksananan

**Affiliations:** 1 Department of Microbiology, Faculty of Medicine, Khon Kaen University, Khon Kaen, Thailand; 2 HPV & EBV and Carcinogenesis Research Group, Khon Kaen University, Khon Kaen, Thailand; 3 Department of Pathology, Cancer Center Amsterdam, VU University Medical Center, Amsterdam, The Netherlands; 4 Department of Medical Oncology, Cancer Center Amsterdam, VU University Medical Center, Amsterdam, The Netherlands; Virginia Commonwealth University, UNITED STATES

## Abstract

Different types of cells infected with Epstein-Barr virus (EBV) can release exosomes containing viral components that functionally affect neighboring cells. Previously, we found that EBV was localized mostly in infiltrating lymphocytes within the stromal layer of cervical lesions. In this study, we aimed to determine effects of exosome-transferred EBV-encoded RNAs (EBERs) on keratinocytes expressing human papillomavirus (HPV) 16 E6/E7 (DonorI-HPV16 HFKs). Lipid transfection of *in vitro*-transcribed EBER1 molecules (ivt EBER1) into DonorI-HPV16 HFKs caused strong induction of interferon (IFN)-related genes and interleukin 6 (IL-6). To gain insights into the physiological situation, monocyte-derived dendritic cells (moDCs), low passage DonorI-HPV16 HFKs and primary keratinocytes were used as recipient cells for internalization of exosomes from wild-type EBV (wt EBV) or B95-8 EBV-infected lymphoblastoid cell lines (LCLs). qRT-PCR was used to determine the expression of EBER1, HPV16 E6/E7, IFN-related genes and IL-6 in recipient cells. The secretion of inflammatory cytokines was investigated using cytometric bead array. Wt EBV-modified exosomes induced both IFN-related genes and IL-6 upon uptake into moDCs, while exosomes from B95-8 EBV LCLs induced only IL-6 in moDCs. Internalization of EBV–modified exosomes was demonstrated in DonorI-HPV16 HFKs, yielding only EBER1 but not EBER2. However, EBER1 transferred by exosomes did not induce IFN-related genes or IL-6 expression and inflammatory cytokine secretion in DonorI-HPV16 HFKs and primary keratinocytes. EBER1 copy numbers in exosomes from wt EBV-infected LCLs were 10-fold higher than in exosomes from B95-8 LCLs (equal cell equivalent), whereas ivt EBER1 was used at approximately 100-fold higher concentration than in exosomes. These results demonstrated that the induction of IFN-related genes and IL-6 by EBER1 depends on quantity of EBER1 and type of recipient cells. High levels of EBER1 in cervical cells or infiltrating dendritic cells may play a role in the inflammation-to-oncogenesis transition of HPV-associated cervical cancer through modulation of innate immune signals.

## Introduction

Epstein-Barr virus (EBV) is a DNA gammaherpesvirus which can establish latent infection in host cells. It enters the human host via a mucosal route, infects and induces proliferation of latently-infected cells. Common tropisms of EBV are B-cells and epithelial cells. Infection of a B-cell by EBV leads to EBV latent gene expression which drives B-cell proliferation [1]. Down regulation of EBV gene expression causes infected B-cells to be invisible to the immune response: thus EBV can persist as a life-long infection in memory B-cells. Infectious virions of EBV are produced in differentiated plasma B-cells and mucosal epithelial cells. Approximately 90% of the world’s population are infected with EBV but most of the infections are asymptomatic or produce nonspecific symptoms [2]. Despite its predominantly benign character, EBV is classified as a group 1 carcinogen according to the International Agency for Research on Cancer (IARC). It is causally associated with a wide range of malignancies including Burkitt lymphoma, Hodgkin lymphoma, nasopharyngeal carcinoma and gastric carcinoma [2].

Cervical cancer is the fourth most common cancer in women worldwide. IARC reported that there were 528,000 new cervical cancer patients and 266,000 deaths from this cancer in 2012 [3]. Human papillomavirus (HPV) persistent infection is a significant risk, although not sufficient, to cause cervical carcinogenesis. Approximately 90% of HPV-infected women clear the infection within a few years [4]. Several cofactors in cervical carcinogenesis have been identified by epidemiological and experimental studies. These cofactors can be classified into 3 groups; 1) environmental or exogenous cofactors such as oral contraceptives, smoking, multiple full-term pregnancies, and concomitant infection with other pathogens; 2) viral cofactors such as specific HPV types, viral load, and integration; 3) host cofactors such as hormones, genetic factors, and immune response status [5].

Our previous study demonstrated that co-occurrence of HPV and EBV DNA in cervical tissues was more frequent in high-grade lesions and squamous cervical-cell carcinoma (SCC) when compared to low-grade lesions and tissues without dysplasia. Moreover, a correlation between EBV infection and high-risk HPV types and HPV episomal form was found. Interestingly, we demonstrated that EBV localized in infiltrating leukocytes adjacent to tumor epithelium and we did not observe EBV infection in epithelial cells [6]. Thus, we hypothesized that HPV infection, which induces many inflammatory signals [7], recruits EBV-carrying B lymphocytes to the infected area. The presence of EBV may provide signals causing the modulation of HPV-infected cells or microenvironment and contributing to tumorigenesis.

EBV proteins, small RNAs and microRNAs (miRNAs) are secreted from EBV- infected cells via exosomes [8–14]. Exosomes are small extracellular vesicles derived from multivesicular bodies (MVBs) or late endosomes participating in intercellular communication. Exosomes range in diameter from 30–100 nm and can be found in many types of body fluids. Epstein-Barr virus-encoded RNAs (EBERs) are small non-coding RNAs abundantly expressed in EBV-infected cells and are secreted in exosomes [14]. These transcripts promote innate immunity modulation and cell growth [15]. Retinoic acid-inducible gene I (RIG-I) and Toll-like receptor 3 (TLR-3) are double-stranded RNA sensors that interact with EBERs and induce downstream signaling pathways including phosphorylation of interferon regulatory factor 3 (IRF-3) and release of interleukins 6 and 10 (IL-6, -10) which act as cellular growth factors [16, 17]. EBERs were also reported to induce type 1 interferons (IFNs), which are antiviral cytokines. Within the EBV-infected B-cells, EBERs counteract the effects of IFNs by blocking protein kinase R (PKR) activity, which is required for the IFN-mediated antiviral effect [18, 19]. EBERs may enhance efficiency of EBV-driven B-cell transformation [20], but overall their role in oncogenesis is poorly understood.

This study aimed to determine the effect of EBERs on keratinocytes expressing HPV16 E6/E7 and monocyte-derived dendritic cells (moDCs), in order to explore potential co-factor effects in HPV-driven cervical carcinogenesis. We demonstrate that exogenous delivery of high levels of EBER1 by lipid transfection induces the expression of IFN-related genes and inflammatory cytokine gene in keratinocytes expressing HPV16 E6/E7, while EBER1 taken in via exosomes does not. Exosomes from wild-type (wt) EBV-infected lymphoblastoid cell lines (LCLs) have an effect on IFN-related gene and inflammatory cytokine gene expression in moDCs. Determination of EBER1 copy numbers revealed that the amount of *in vitro*-transcribed EBER1 (ivt EBER1) transfected into cells was significantly higher than EBER1 in exosomes. The results suggest that the induction of IFN-related genes and inflammatory cytokine genes by EBER1 depends on the number of copies of EBER1 and the type of recipient cell.

## Materials and Methods

### Cell lines

EBV-infected LCLs used were RN LCLs, which contain EBV B95-8 strain, and IK140508 LCLs which contain wt EBV. BJAB cells, which are EBV-negative diffuse large B-cell lymphoma, were used as negative controls. Human foreskin keratinocytes (HFKs) used in this study as recipient cells included DonorI-HPV16 HFKs (HFKs transduced with recombinant retrovirus containing E6/E7 open reading frame, passages 19–32) [21], FK16A cells (HFKs transfected with full-length HPV16 genome) [22] and primary HFKs. All primary cells were obtained from donors who have given informed consent approved by the institutional review board of the VU University Medical Center (VUmc). The institutional review board of the VUmc did not need to specifically approve this study as we used left-over human materials from circumcisions and volunteers blood cell specimens, which is covered by the general “good research practice” regulations within VUmc. However, this study is one part of our project entitled “Co-infection between EBV & HPV and mechanisms of cervical carcinogenesis” which was approved by the Khon Kaen University Ethics Committee in human research (no. HE561264). Foreskins from which primary keratinocytes were isolated, were obtained and used in an anonymous fashion in accordance with the "Code for Proper Secondary Use of Human Tissues in the Netherlands" as formulated by the Dutch Federation of Medical Scientific Organizations (http://www.fmwv.nl and www.federa.org). Parents of circumcised boys orally consented to this secondary use (www.besnijdeniscentrum.nl). This study followed the ethical guidelines of the Institutional Review Board of the VUmc. LCLs and BJAB cells were cultured in RPMI medium (Lonza, Basel, Switzerland) supplemented with 10% fetal bovine serum (FBS) (HyClone, Utah, USA), 100 U/ml penicillin G, 100 μg/ml streptomycin sulfate, and 2 mM glutamine (PSG). All keratinocytes were cultured in Keratinocyte-SFM medium with L-glutamine, epidermal growth factor (EGF) and Bovine Pituitary Extract (BPE) (Gibco, Massachusetts, USA) with PSG.

### Establishment of moDCs

Peripheral blood mononuclear cells (PBMCs) were isolated from whole blood of healthy donors using Lymphoprep (Stemcell Technologies, British Columbia, Canada) according to the manufacturer’s protocol. All donors gave written informed consent for scientific use approved by the institutional review board of the VUmc. The participant consent was asked from the volunteer to sign in the informed consent form. This consent procedure was approved by the institutional review board of the VUmc. CD14+ monocytes were isolated using CD14 MicroBeads, Human (Miltenyi Biotec, Bergisch Gladbach, Germany) and cultured in IMDM medium (Lonza, Basel, Switzerland) with 10% FBS and PSG with 0.01 ng/mL of IL-4 and 1,000 units/mL of GM-CSF in a cell-culture incubator at 37°C, 5% CO_2_ for 5 days.

### Cell proliferation assay

Cell proliferation was measured using a cell proliferation kit I (MTT) (Roche, Basel, Switzerland) according to the manufacturer’s protocol. One-hundred microliters of cells were plated in 96-well plates (10^3^ cells/well) and incubated at 37°C in a cell-culture incubator for 0, 24, 48, 72, 96 and 120 hours. The intensity of color at 550 and 650 nm was measured using an ELISA reader. The percentage of viable cells was calculated using the following formula: viable cells (%) = (OD of treated sample/OD of untreated sample) × 100.

### Exosome isolation

Exosomes from EBV-infected LCLs and BJAB cells were isolated by differential ultracentrifugation. The cells were washed by centrifugation at 1,100 rpm for 5 minutes and recultured at a density of 0.5 x 10^6^ cells/mL in RPMI-1640, supplemented with 5% exosome-depleted FBS and PSG for 48 hours. The viability of cells was checked before supernatant collection and found to be at least 95%. Percentage of cell death was less than 5%. Cell culture medium containing 100 x 10^6^ cells was centrifuged twice at 500 x g for 10 minutes at 4°C to remove the cells. After the second centrifugation, the supernatant was centrifuged twice at 2,000 x g for 15 minutes at 4°C to remove cell debris. The supernatant could be stored at -80°C or used immediately for the next step. The supernatant was centrifuged again twice at 10,000 x g for 30 minutes with slow braking at 4°C. To pellet the exosomes, the supernatant was centrifuged at 70,000 x g for 60 minutes with slow braking at 4°C. The supernatant was carefully removed from the exosome pellet and the pellet was pooled together with additional phosphate buffer saline (PBS). The exosome suspension was centrifuged again at 70,000 x g for 60 minutes with no braking at 4°C. The supernatant was carefully removed without disturbing the exosome pellet, which was then resuspended in 200 μL PBS and stored at -80°C.

### Western blotting

CD63 protein was used as an exosomal marker and cytochrome C protein was used as a cellular marker (14). Primary antibodies used in the studies included purified mouse anti-human CD63 diluted 1:250 (BD Pharmingen, New Jersey, USA) under non-reducing conditions as recommended by the manufacturer and purified mouse anti-cytochrome C diluted 1:250 (BD Pharmingen, New Jersey, USA) under reducing conditions. Equal amounts of exosome suspension and loading dye were mixed, incubated at 95°C for 5 minutes and immediately placed on ice for 5 minutes before loading on a sodium dodecyl sulfate-polyacrylamide gel for electrophoresis (SDS-PAGE). The protein on the gel was transferred to nitrocellulose blotting membrane (Amersham Protran, Little Chalfont, UK). Signal detection was performed using Enhanced Chemiluminescence (ECL) prime Western Blotting Detection Reagent (Amersham, Little Chalfont, UK) according to the manufacturer’s instruction. The membrane was exposed to X-ray film for visualization.

### Labelling of exosomes with PKH67 green fluorescent linker dye

Exosomes in 1x PBS were labelled with green fluorescent dye using a PKH67 green fluorescent cell linker mini kit for general cell membrane labeling (Sigma-Aldrich, Missouri, USA) according to manufacturer’s protocol. After labeling, the exosome mixture was transferred to an ultracentrifuge tube and centrifuged at 70,000 x g for 1 hour with no braking at 4°C. The supernatant was carefully removed by pipetting without disturbing the exosome pellet, which was resuspended in 1x PBS and stored at -80°C.

### EBER1 *in vitro* transcription and transfection

DNA of plasmid pcDNA3 containing full-length EBER1 was cut with *Xba*I restriction enzyme (Roche, Basel, Switzerland). The linearized pcDNA3-EBER1 was extracted from a gel using Qiaex II gel extraction kit (Qiagen, Hilden, Germany), according to the manufacturer’s protocol, and eluted in RNase-free water. To perform *in vitro* transcription, the reaction mixture contained 1 μg of linearized pcDNA3-EBER1, 1x transcription buffer, 0.5 mM rNTPs, 10 mM DTT, 24 units RNAsin, 126 units T7 RNA polymerase (Promega, Wisconsin, USA) and RNase-free water to 120 μL. The reaction mixture was incubated at 37°C for 3 hours and subjected to DNase treatment using RNase-free DNase (Promega, Wisconsin, USA) according to the manufacturer’s protocol. The ivt EBER1 was purified using TRIzol^®^ reagent (Invitrogen, California, USA) according to manufacturer’s protocol. Ivt EBER1 was transfected into DonorI-HPV16 HFKs using Lipofectamine 2000 (ThermoFisher Scientific, Massachusetts, USA). One-hundred nanograms of ivt EBER1 was mixed with 100 μL cell culture medium without any antibiotics and vortexed for 15 seconds. Lipofectamine 2000 was added (1 μL: 3 μg RNA), vortexed and incubated at room temperature for 30 minutes. Two hundred microliters of cell culture medium without antibiotics was added and the mixture was added to cells in a 24-well plate. The cells were incubated with the transfection mixture for 4 hours in a cell culture incubator and refreshed with new medium containing antibiotics.

### Detection of EBERs

RNA from cells and exosomes was isolated using TRIzol^®^ reagent (Invitrogen, California, USA) according to the manufacturer’s protocol. The RNA pellet was resuspended in 10 μL of RNase-free water. The amount, quality, and composition of isolated RNA were analyzed using the NanoDrop 2000c spectrophotometer (ThermoFisher Scientific, Massachusetts, USA). cDNA was synthesized using TaqMan MicroRNA Reverse Transcription Kit (ThermoFisher Scientific, Massachusetts, USA) according to the manufacturer’s protocol with 125 nM for each stem-loop primer. The stem-loop primer sequences are shown in Supporting information: S1 Table. qRT-PCR was performed using LightCycler^®^ 480 SYBR green I master (Roche, Basel, Switzerland) according to the manufacturer’s protocol. The primer sequences and concentration used in this study are shown in Supporting information: S2 Table. All samples were run in duplicate using the LightCycler^®^ 480 Instrument (Roche, Basel, Switzerland).

### EBER1 quantification

The RNA pellet extracted by TRIzol^®^ reagent (Invitrogen, California, USA) was resuspended in RNase-free water and subjected to DNase treatment with RQ1 RNase-free DNase (Promega, Wisconsin, USA). To precipitate RNA, the reaction mix (26.5 μL in total) contained 1 μL of 3 M NaAc pH 5.3, 25 μL of absolute ethanol and 0.5 μL of linear acrylamide. The reaction mix were added to DNase-treated RNA solution and incubated at -80°C for 1 hour. Then centrifugation at 12,000 x g for 30 minutes at 4°C was performed to precipitate RNA. After removal of the supernatant, 500 μL of 70% cold ethanol was added and centrifugation repeated at 12,000 x g for 5 minutes at 4°C. The supernatant was removed and the pellet was dried at room temperature. cDNA was synthesized from RNA template using 2 μM of EBER1.1 and RNY1 stem-loop primer. The primer sequences and concentrations used in this study are shown in Supporting information: S3 Table. When determining EBER copy number using quantitative real-time PCR, pCR-BluntII-TOPO containing full-length EBER1 was used for constructing the standard curve. All samples were run in duplicate using the LightCycler^®^ 480 Instrument (Roche, Basel, Switzerland).

### Determination of HPV oncogene expression using conventional PCR

RT-PCR was performed using HPV16 E6-specific primers to amplify nucleotides 204–525, which allows the detection of full-length E6 transcripts and spliced E6*I mRNA [22]. cDNA was synthesized using AMV reverse transcriptase (Promega, Wisconsin, USA), according to the manufacturer’s protocol, with 1.25 μM of HPV16E6502as primer and 1.25 μM MP-GAPDH reverse primer. The 25 μL PCR reaction contained 1x PCR buffer (ThermoFisher Scientific, Massachusetts, USA), 0.1 mM dNTP mix, 0.5 μM forward primer (HPV16E6204s or MP-GAPDH-F), 0.5 μM reverse primer (HPV16E6502as or MP-GAPDH-R), 0.5 unit of AmpliTaq Gold DNA polymerase (ThermoFisher Scientific, Massachusetts, USA), 2 μL of cDNA template and DNase-free water to 25 μL. Amplification was done with the following parameters; initial denaturing at 95°C for 4 minutes; 30 cycles comprising of 95°C for 30 seconds, 60°C for 1 minutes, 72°C for 90 seconds; and final extension at 72°C for 4 min. PCR products were detected by electrophoresis in a 2% agarose gel and stained with ethidium bromide. The primer sequences used in this study are shown in Supporting information: S2 Table.

### Determination of HPV oncogene expression using TaqMan qRT-PCR

A quantitative RT-PCR for E7 was performed on DNase-treated RNA [23] using RQ1 DNase (Promega, Wisconsin, USA) according to the manufacturer’s protocol. cDNA synthesis was performed after DNase treatment using 1.25 μM of specific reverse primers (qE7_rv_6 and snRNP828R). To control for DNA contamination, a reaction without AMV reverse transcriptase (minus-RT control) was included each time. TaqMan qRT-PCR for HPV16 E7 quantification was performed as described previously [23]. Relative expression was calculated using the 2^- ddCt^ method with small nuclear ribonucleic protein (snRNP) as a calibrator gene. The primer and probe sequences used in this study are shown in Supporting information: S2 Table.

### Determination of IFN-related gene and IL-6 expression

RNA from recipient cells was isolated using TRIzol^®^ reagent (Invitrogen, California, USA) according to the manufacturer’s protocol. The RNA pellet was resuspended in 10 μL of RNase-free water. The amount, quality and composition of the RNA isolated were analyzed using a NanoDrop 2000c spectrophotometer (ThermoFisher Scientific, Massachusetts, USA). cDNA was synthesized using the Reverse Transcription System (Promega, Wisconsin, USA) according to the manufacturer’s protocol with 1 μg of RNA template. SYBR green qRT-PCR was performed using the LightCycler^®^ 480 SYBR green I master (Roche, Basel, Switzerland). The primer sequences and concentrations used in this study are shown in Supporting information: S2 Table. All samples were run in duplicate using the LightCycler^®^ 480 Instrument (Roche, Basel, Switzerland). Relative expression was calculated using the 2^-ddCp^ method and glyceraldehyde 3-phosphate dehydrogenase (GAPDH) as a calibrator gene.

### Determination of inflammatory cytokine secretion in cell culture supernatant

Secretion of inflammatory cytokines was determined using a BD™ CBA Human Inflammatory Cytokines Kit (BD Biosciences, California, USA) according to the manufacturer’s protocol. The kit was used to quantitatively measure IL-1β, IL-6, and tumor necrosis factor (TNF) protein levels.

### Statistical analysis

Relative expression of genes was calculated using the 2^- ddCp^ method and GAPDH or snRNP as a calibrator gene. Data were analyzed by one-way analysis of variance (ANOVA) with Tukey tests and presented as means with standard error of the mean (SEM). A value of P < 0.05 was considered statistically significant.

## Results

### *In vitro* transcribed EBER1 induces IFN response in DonorI-HPV16 HFKs

**Fig 1:**
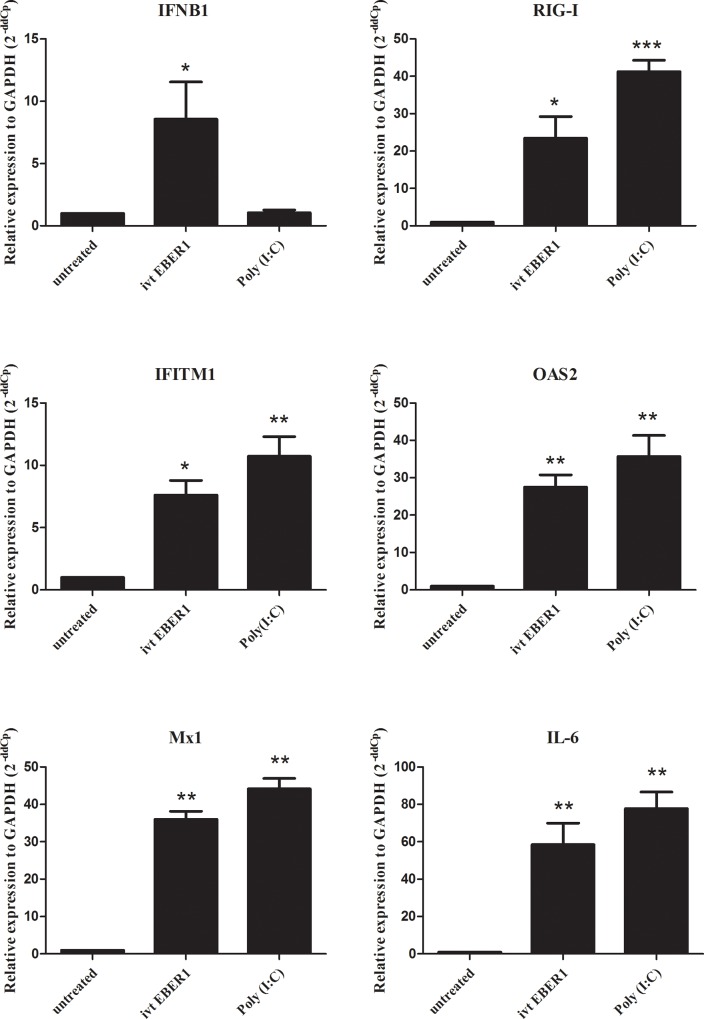
IFN-related gene and IL-6 expression in DonorI-HPV16 HFKs in the presence of lipid delivered ivt EBER1. One hundred ng of ivt EBER1 was transfected into DonorI-HPV16 HFKs and incubated for 24 hours. Gene expression levels were determined by qRT-PCR. Untreated (control) cells were transfected with lipofectamine alone. Relative expression level was corrected with the housekeeping gene GAPDH (2^-ddCp^). *; P < 0.05, **; P < 0.01, ***; P < 0.001.

To investigate the effect of EBER1 in HFKs expressing HPV16 E6/E7 (DonorI-HPV16 HFKs), we generated EBER1 molecules by *in vitro* transcription [24] and introduced them into DonorI-HPV16 HFKs by lipid transfection and incubated the cells for 24 hours. IFN-related mRNA levels were determined using qRT-PCR. Target genes were selected from high-density microarray data of moDCs incubated with EBV-modified exosomes for 18 hours. The chosen targets were IFNβ-1, RIG-I, interferon-induced transmembrane protein 1 (IFITM1), 2′-5′ ligoadenylate synthetase (OAS2), interferon-induced GTP-binding protein Mx1 (Mx1) and proinflammatory cytokine IL-6 as recently published [24]. The results showed that all five IFN-related genes and IL-6 were strongly induced by lipid delivery of EBER1 transcripts (Fig 1). This suggests that delivery of EBER1 via lipid transfection could trigger viral sensors in DonorI-HPV16 HFKs to induce IFN-related gene expression.

### Analysis of exosomes secreted from EBV-infected LCLs

**Fig 2:**
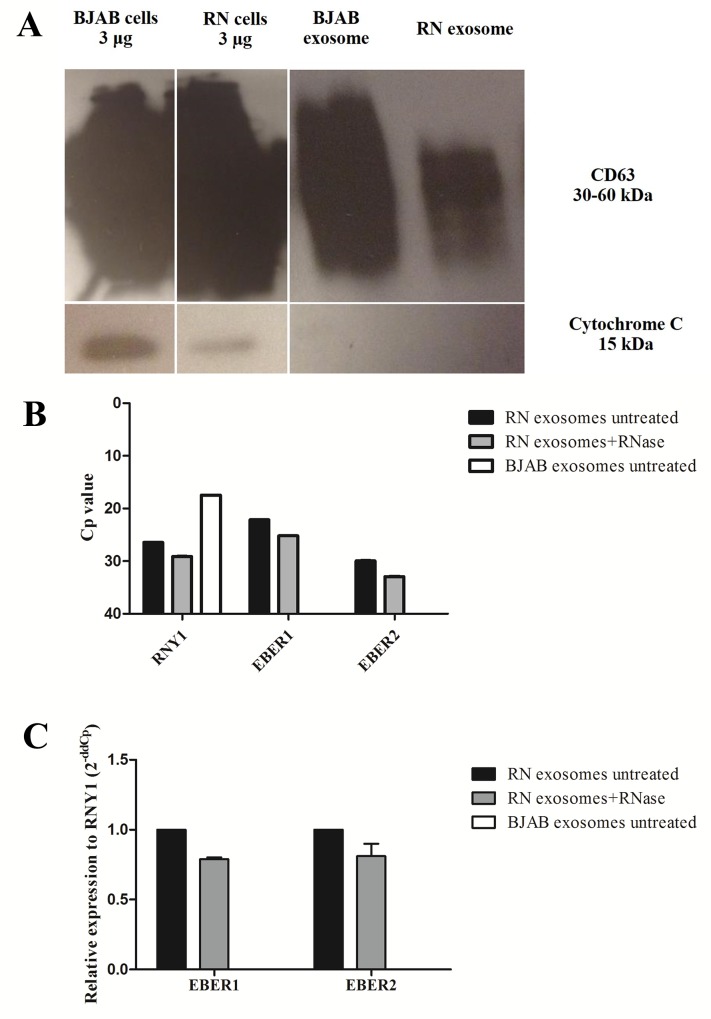
Exosomes isolated from EBV-infected LCLs. (A) CD63 was used as the exosomal marker and cytochrome C was used to determine cellular contamination. (B) Cp values of EBERs and RNY1 (housekeeping gene) in exosomal fractions from equal cell equivalents of untreated and RNase A-treated RN and untreated BJAB exosomes, as determined by stem-loop RT-PCR. (C) Relative expression level of EBERs in untreated RN exosomes compared to RNase A-treated RN exosomes, corrected with housekeeping gene RNY1 (2^-ddCp^). BJAB exosomes were used in a negative control. Three batches of exosomes were used for determination.

To gain insight into the physiological situation, exosomes from cell culture supernatant of EBV-infected LCLs (EBV-modified exosomes) and BJAB cells were isolated using differential ultracentrifugation and subjected to protein extraction. Exosomal protein was used for detection of the exosomal marker (CD63) and cellular component contamination (cytochrome C) was examined using western blotting. The exosomal marker, CD63, was found in the purified exosomes, but cytochrome C was not found. This result indicated that there was no contamination from cellular components in the vesicle pellet, as shown in Fig 2A. To investigate whether EBERs were confined inside the exosomes from EBV-infected LCLs, isolated exosomes from RN cells were subjected to RNase A treatment (0.4 μL/mL at 37°C for 1 hour) before RNA extraction in order to degrade the nucleic acid outside exosomes. The extracted RNA was used for determination of EBER1 and EBER2 by stem-loop RT-PCR. As shown in Fig 2B, the Cp values of EBERs were slightly increased when RN exosomes were treated with RNase A, which indicates a lower level of EBERs in RNase A-treated exosomes. Levels of EBER2 in the exosomal fraction were lower than those of EBER1 (Fig 2B). The relative expression levels of EBERs were slightly lower when RN exosomes were treated with RNase A (Fig 2C). EBERs were not found in exosomes from BJAB cells. The results suggested that the majority of EBERs were protected within the exosomal membrane and were not susceptible for RNA degradation.

### Wt EBV-modified exosomes induce IFN-related genes and IL-6 expression in moDCs

**Fig 3:**
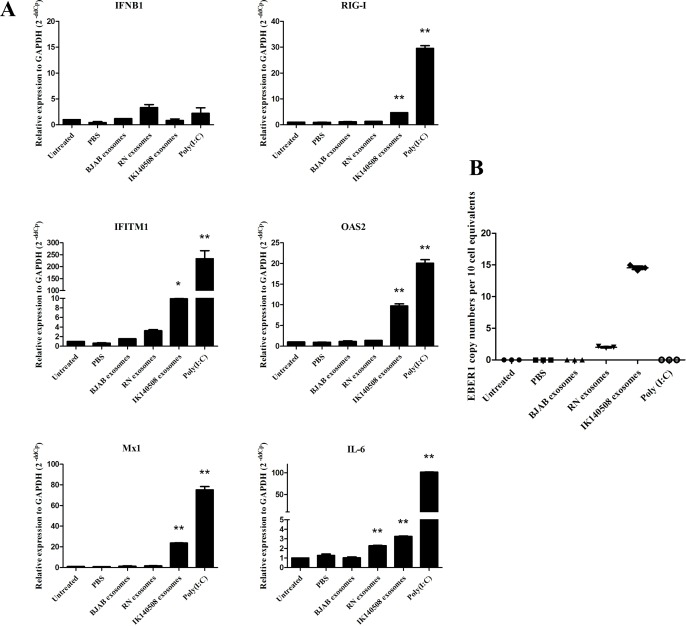
Induction of IFN-related genes and IL-6 expression in moDCs. (A) EBV-modified exosomes were added to moDCs and incubated for 24 hours. The expression levels of IFN-related genes and IL-6 were determined by qRT-PCR. Relative expression level was corrected with the housekeeping gene GAPDH (2^-ddCp^). *: P < 0.05; **: P < 0.01. (B) EBER1 copy number in moDCs per 10 cell equivalents. EBER1 copy number in moDCs was determined by qRT-PCR using a plasmid containing full-length EBER1 for standard curve calibration.

Dendritic cells (DCs) are antigen presenting cells (APCs) which reside in the stromal layer of tissues and also in squamous epithelia (Langerhans cells). DCs acquire extracellular antigen by receptor-mediated endocytosis, macropinocytosis or phagocytosis. Exosomes are a source of antigen for APCs and are internalized and transferred functional signals into dendritic cells [10, 24, 25]. To test whether EBER1-containing exosomes influence the expression of IFN-related genes and IL-6 in DCs, we incubated moDCs from PBMCs with purified exosomes from EBV-infected LCLs for 24 hours. The results showed that RIG-I, IFITM1, OAS2, Mx1 and IL-6 were induced by IK140508 exosomes whereas RN exosomes had an effect only on the induction of IL-6 but not IFN-related genes in moDCs (Fig 3A). The different capacities of RN and IK140508 exosomes to induce IFN-related genes and IL-6 led us to investigate EBER1 copy number transferred via exosomes into recipient cells. qRT-PCR was performed to determined EBER1 copy numbers using a plasmid containing full-length EBER1 for standard curve establishment. The results demonstrated that the copy number of EBER1 transferred via exosomes from IK140508 cells was 7-fold higher than those transferred from RN exosomes (Fig 3B). This may cause the different induction of target genes in moDCs.

### Internalization of EBV-modified exosomes into HPV16 E6/E7 expressing HFKs and stability of EBER1 transferred via exosomes into the recipient cells

**Fig 4:**
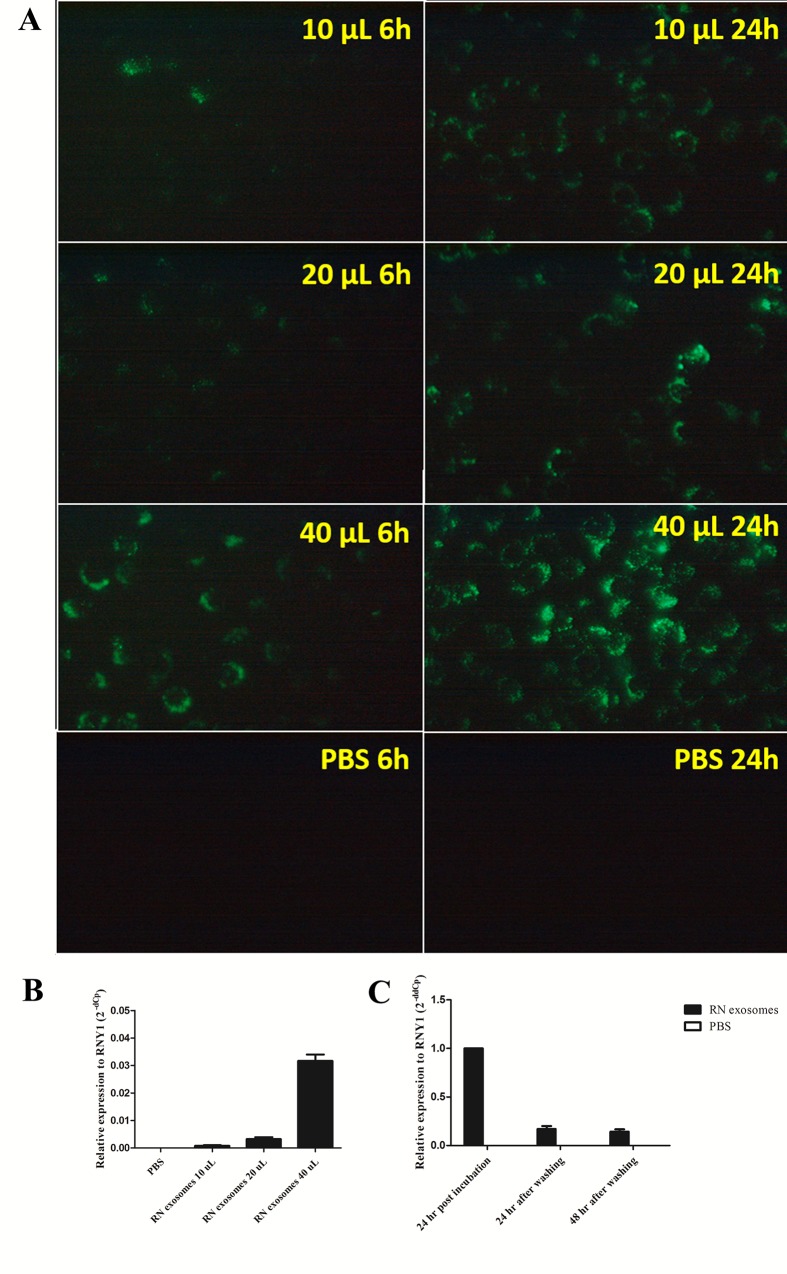
Internalization of exosomes into DonorI-HPV16 HFKs. (A) DonorI-HPV16 HFKs were incubated with exosomes labelled with fluorescent dye PKH67 in 10, 20 and 40 μL of exosome suspension and observed for PKH67 positive cells at 6 and 24 hours post incubation. (B) Levels of EBER1 in DonorI-HPV16 HFKs after incubation with 10, 20 or 40 μL of RN exosomes for 24 hours. (C) Stability of EBER1 in DonorI-HPV16 HFKs. DonorI-HPV16 HFKs were incubated with RN exosomes for 24 hours. The cells were then washed and cultured for an additional 24 and 48 hours in new medium without RN exosomes. Levels of EBER1 were measured using stem-loop real-time RT-PCR.

As shown in Fig 3A, EBV-modified exosomes could induce the expression of EBER1 target genes in moDCs. This indicated that EBV-modified exosomes, which contained EBER1, were internalized and the EBER1 could function in the moDCs. Next, to determine whether exosomes can be internalized by HFKs (non-APC cells) expressing HPV16 E6/E7, DonorI-HPV16 HFKs were incubated with various quantities of RN exosomes labeled with green fluorescent dye PKH67 and observed for PKH67 positive cells at 6 and 24 hours post incubation. Increasing time and quantity of exosomes led to more and brighter fluorescent cells (Fig 4A), indicating specific uptake. The results indicated that EBV-modified exosomes could be internalized by DonorI-HPV16 HFKs in a dose- and time-dependent manner. To investigate the transfer of EBERs into DonorI-HPV16 HFKs, stem-loop RT-PCR was performed. After 24 hours of incubation with RN exosomes, DNA and RNA were extracted from DonorI-HPV16 HFKs. Quantity of EBER1 increased when increased quantities of exosomes were used (Fig 4B). However, EBER2 and EBV DNA were not found in recipient cells (data not shown). To investigate the stability of EBERs transferred via exosomes into the recipient cells. RN exosomes were incubated with DonorI-HPV16 HFKs. After 24 hours of incubation, cell culture supernatant was removed and the cells were washed with PBS to remove unbound exosomes. New medium was added and the cells were cultured for an additional 24 and 48 hours. EBER1 levels were determined using stem-loop real-time RT-PCR. As shown in Fig 4C, the initial EBER1 level dramatically decreased by 24 hours after washing the cells and the level remained stable until 48 hours after washing.

### EBV-modified exosomes have no effect on HPV oncogene expression or proliferation of DonorI-HPV16 HFKs

**Fig 5:**
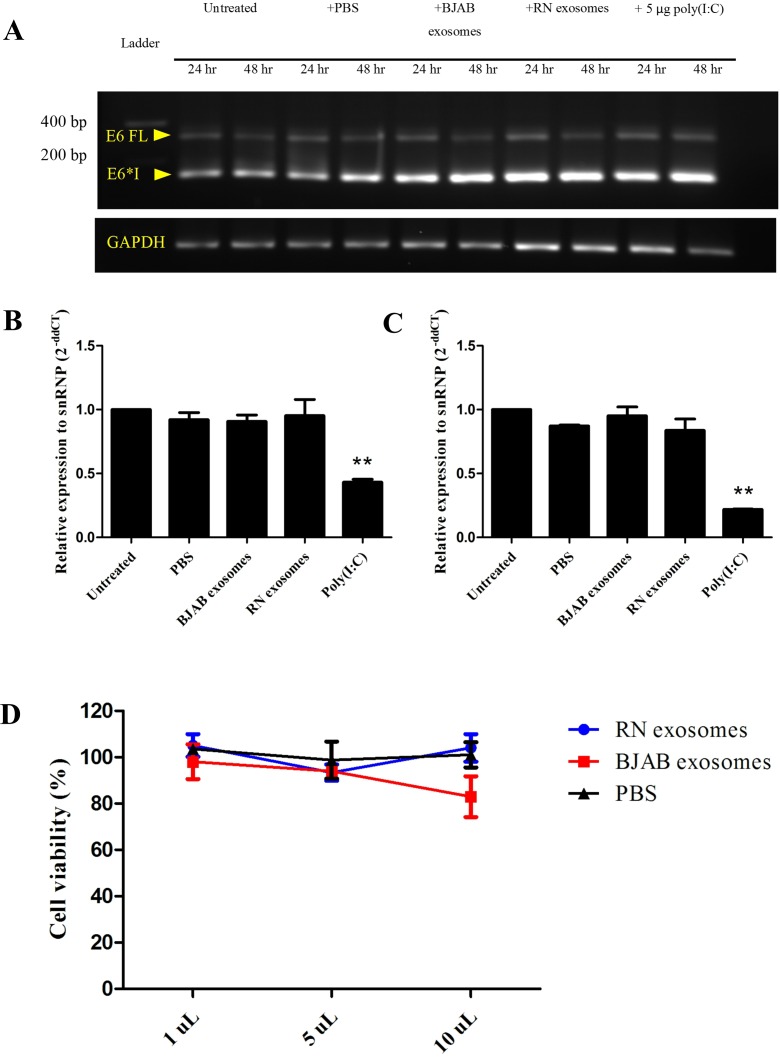
HPV16 E6E7 mRNA expression after incubation with exosomes. (A) E6 FL indicates full length mRNA (321 nucleotides) and E6*I indicates spliced transcripts (138 nucleotides). GAPDH (140 nucleotides) served as a house-keeping control. (B) Relative E7 mRNA expression in DonorI-HPV16 HFKs and (C) FK16A. mRNA expression was determined by qRT-PCR after 24 hours of incubation. Level of expression was normalized relative to the housekeeping gene snRNP. **: P < 0.01. (D) The effect of exosomes on cell growth of DonorI-HPV16 HFKs. DonorI-HPV16 HFKs were treated with 1.0, 5.0 or 10.0 μL of PBS, RN exosomes and BJAB exosomes for 120 hours. Cell viability was monitored using the MTT assay.

We firstly investigated the effect of EBV-modified exosomes on HPV oncogene expression in recipient cells using RT-PCR. The purified exosomes from RN or BJAB cells were incubated with DonorI-HPV16 HFKs for 24 and 48 hours. RT-PCR was performed on cDNA samples using HPV16 E6-specific primers, which allows the detection of full-length E6 transcripts and spliced E6*I mRNA. As shown in Fig 5A, a full length E6 transcript was observed. After 48 hours of incubation, the quantity of this transcript was slightly lower than at 24 hours. The main product expressed in DonorI-HPV16 HFKs is the E6*I splice variant. BJAB and RN exosomes and poly (I:C) seemed to have a slight effect on the splicing of HPV16 E6E7. Therefore, we confirmed the expression of HPV oncogenes using Taqman quantitative PCR for E7. Neither treatment with BJAB nor with RN exosomes for 24 hours had an effect on HPV16 oncogene expression. Interestingly, treatment with poly (I:C) decreased the expression of HPV16 E7 (Fig 5B). We also confirmed the results in HFK cells transfected with full-length HPV16 (FK16A) containing the real promoter of HPV16 E6/E7: no induction of E6/E7 was observed (Fig 5C). These results indicated that EBV-modified exosomes had no effect on HPV oncogene expression. We also confirmed whether EBV-modified exosomes have an effect on cell proliferation of DonorI-HPV16 HFKs using the MTT assay. The volumes of RN exosomes added to the recipient cells were 1.0, 5.0 and 10.0 μL. BJAB exosomes and PBS were used as negative controls. Compared to the untreated cells (taken as 100% viable), different quantities of RN exosomes and PBS did not show any effect on DonorI-HPV16 cell growth (Fig 5D). However, exposure to 10 μL of BJAB exosomes caused a decrease in cell viability from 100% to 82.9% (Fig 5D).

### EBER1 from EBV-modified exosomes has no effect on IFN and proinflammatory cytokine responses in HFKs

**Fig 6:**
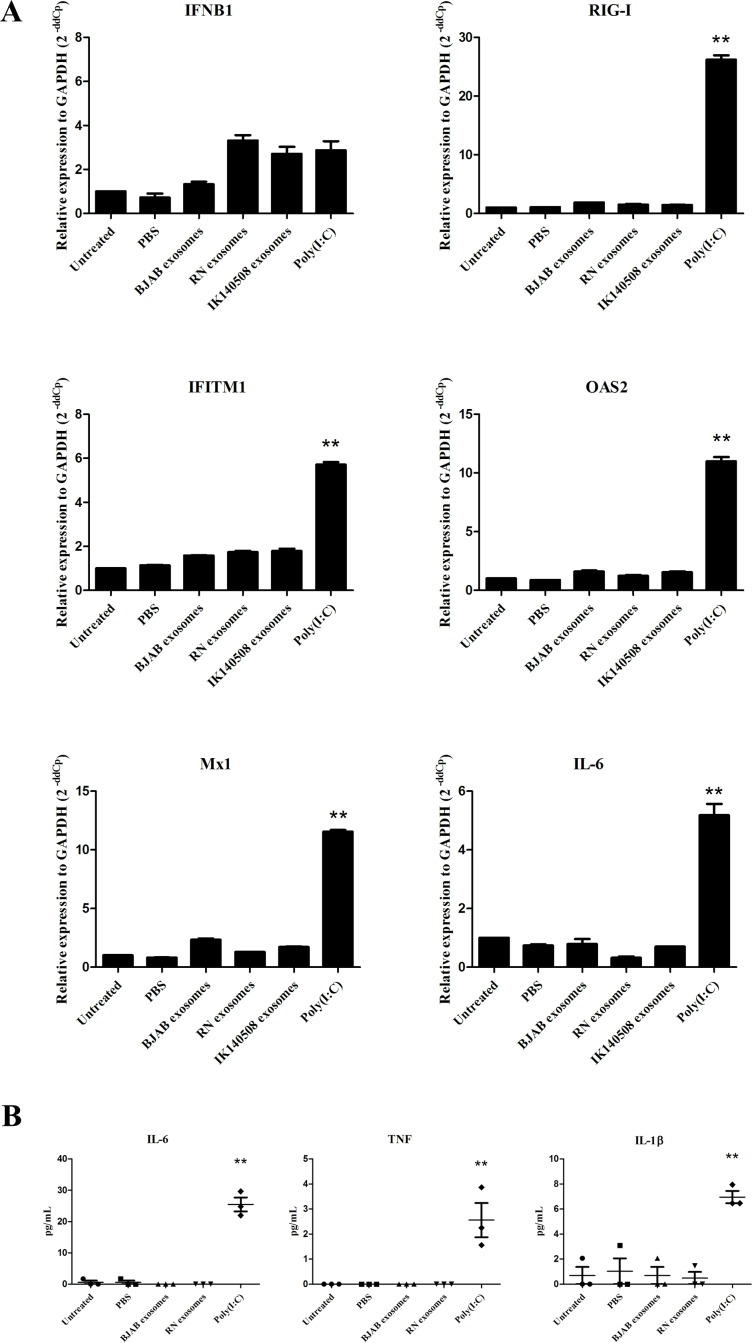
IFN-related genes and IL-6 expression and the production of proinflammatory cytokines in DonorI-HPV16 HFKs. (A) EBV-modified exosomes were added to DonorI-HPV16 HFKs and incubated for 24 hours. The expression levels of IFN-related genes were determined by qRT-PCR. Relative expression level was corrected relative to the housekeeping gene, GAPDH (2^-ddCp^). (B) Cytokine secretion from DonorI-HPV16 HFKs after incubation with exosomes for 48 hours. Protein concentration in cell culture supernatant was determined using a cytometric bead array for human inflammatory cytokines. **: P < 0.01.

In order to investigate the influence of EBV-modified exosomes on IFN-related gene and IL-6 expression, DonorI-HPV16 HFKs were incubated with exosomes purified from RN and IK140508 cell-culture supernatant for 24 hours, and IFN-related and IL-6 mRNA levels were determined using qRT-PCR. The results showed that EBV-modified exosomes from both RN and IK140508 cells could not induce the expression of IFN-related genes and IL-6 in DonorI-HPV16 HFKs, but these genes were induced by 5 μg poly (I:C), which was used as a positive control (Fig 6A). The effect of EBV-modified exosomes on HPV-positive HFKs was also investigated in FK16A cells which were HFKs transfected with full-length HPV16. The results were similar to those in DonorI-HPV16 HFKs (S1 Fig). A recent study has shown that proinflammatory cytokine production in HFKs was suppressed by HPV by augmenting the expression of interferon-related development regulator 1 (IFRD1) in a manner dependent on an epidermal growth-factor receptor (EGFR), thus suppressed NFκB activation and resulting in decrease of immune system-driven cytokine expression [26]. To prove that E6/E7 expression in DonorI-HPV16 HFKs suppressed the induction of IFN and a proinflammatory response in the cells after treatment with EBV-modified exosomes, HPV-negative primary HFKs were used in the experiments. After incubation with EBV-modified exosomes for 24 hours, IFN-related and IL-6 gene expression of primary HFKs was determined by qRT-PCR. The results showed that the presence of EBV-modified exosomes did not induce the expression of the genes of interest (S2 Fig). In addition, we also used a cytometric bead array to determine the effects of EBV-modified exosomes on secretion of proinflammatory cytokines (IL-6, TNF and IL-1β) by DonorI-HPV16 HFKs. Protein concentration in cell culture supernatant was determined after incubation with exosomes for 48 hours. Similar to the results in mRNA level, EBV-modified exosomes could not induce the secretion of proinflammatory cytokines in DonorI-HPV16 HFKs (Fig 6B). These results demonstrated that, although EBER1 from exosomes could be detected in the recipient cells, its presence had no effect on the production of IFN-related genes or proinflammatory cytokines in HFKs with and without the presence of HPV16 E6/E7 oncogenes.

### Quantitative EBER1 levels in EBV-modified exosomes

**Fig 7:**
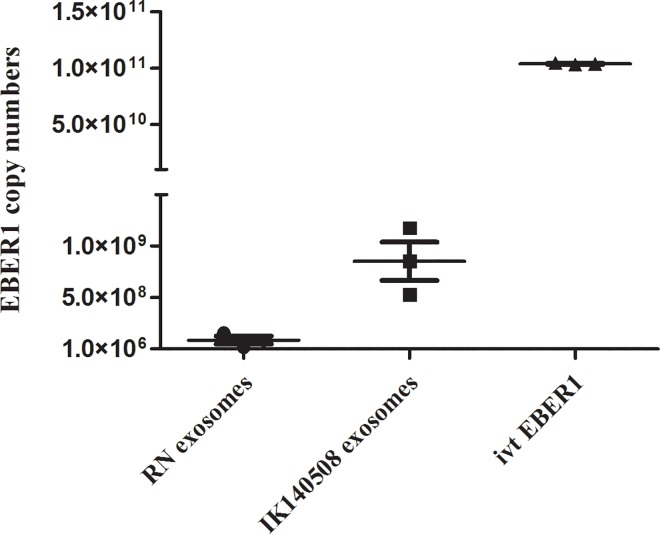
EBER1 copy numbers in the exosomal fraction from 25x10^6^ cells of RN and IK140508 LCLs and 100 ng ivt EBER1. EBER1 copy numbers were determined by qRT-PCR using pCR-BluntII-TOPO containing full-length EBER1 for standard curve establishment.

We wished to determine whether EBER1 copy numbers differ in exosomes from RN or IK140508 LCLs. RNA was extracted from exosomes from 25x10^6^ LCLs, which was the amount used for incubation with recipient cells. One hundred nanograms of ivt EBER1 was also used. Absolute qRT-PCR was performed on DNaseI-treated RNA samples. As shown in Fig 7, EBER1 copy number in exosomes from IK140508 LCLs was 10-fold higher than in RN exosomes. In addition, a huge amount of EBER1 was observed in ivt EBER1 which is approximately 100-fold higher than from IK140508 exosomes.

## Discussion

EBV is one of the most successful viruses, latently persisting in B lymphocytes for the lifetime of the infected host. Although EBV persistence is generally considered benign, EBV can play an important role in an increasing number of acute, chronic and malignant diseases [27–29]. In the last years, exosomes, extracellular vesicles released upon fusion of multivesicular bodies with plasma membrane, have emerged as powerful mediators which can penetrate and change the behavior of neighboring cells. Several lines of evidence demonstrated that EBV-infected B lymphocytes actively secrete exosomes and have biological function in recipient cells [10, 13, 30, 31].

Our previous study showed that EBV-HPV co-infections with high-risk HPV were significantly more frequent in high-grade squamous intraepithelial lesion (HSIL)/SCC groups. The co-infection was associated with HPV episomal form. *In- situ* hybridization staining of EBERs in cervical tissues showed localization of EBV in the nuclei of infiltrating lymphocytes in stromal layers [6]. Thus, we hypothesized that EBV-infected B lymphocytes infiltrating cervical tissues may not be just a commensal agent but may play a role in manipulating the microenvironment and HPV-infected cells to induce the development of cervical cancer by secretion of their products via exosomes.

EBERs are secreted abundantly in exosomes [14], expressed in all three patterns of EBV latency and can be detected by pathogen recognition receptors (PRRs) to induce inflammatory cytokine production in recipient cells [32, 33]. EBERs are recognized by RIG-I which is dsRNA sensor residing in the cytoplasm. The interaction leads to activation of NFκB and IRF3, which in turn induce protective cellular genes, including type I IFNs [33]. It is possible that EBERs activate inflammation via dsRNA sensors, thus contributing to EBV-mediated pathogenesis both in lymphoproliferative diseases and cancers.

In this study, we investigated the function of EBER1 in HFKs expressing HPV16 E6/E7 and in moDCs, a cell type abundantly present in cervical lesions and actively involved in local inflammation and antigen presentation (23). First, to investigate the effect of EBER1 in HFKs expressing HPV16 E6/E7 (DonorI-HPV16 HFKs), we generated EBER1 molecules by *in vitro* transcription [24] and introduced them into DonorI-HPV16 HFKs by lipid transfection. Genes of interest were selected from high-density microarray results after incubation of EBV-modified exosomes with moDCs for 18 hours [24] and included IFNβ-1, RIG-I, IFITM1, OAS2, Mx1 and IL-6. Twenty-four hours after transfection, all five IFN-related genes and IL-6 were strongly induced in these cells, indicating that EBER1 delivered by lipid transfection acts as pathogen-associated molecular pattern (PAMP), triggering double-stranded RNA sensors in the cytoplasm to induce IFN-related genes and proinflammatory cytokine expression. Subsequently we explored EBER1 effects under more physiologically relevant conditions. We purified exosomes from two types of EBV-infected (latency III) LCLs, including a wt and B95-8 strain, using differential ultracentrifugation. We showed that EBER1 and low levels of EBER2 could be detected in exosomal fractions of culture supernatant from both LCL types. In EBV-infected cells, although both EBERs are transcribed at approximately equal rates, EBER1 was found to be present at 10-fold higher levels when compared to EBER2 [34]. Clarke et al. in 1992 reported that EBER1 had a longer half-life than EBER2. They showed that in the presence of actinomycin D, the half-lives of EBER1 and EBER2 are 8 to 9 hours and 45 minutes, respectively [35]. Our results suggested that levels of EBERs loaded into exosomes may be related to levels of EBERs in the cells. Treatment of purified exosomes with RNase A prior to RNA extraction slightly reduced EBER positivity, indicating that some EBER molecules passively associate with the surface of exosomes. However, the majority of EBERs in the exosomal fraction are protected from RNase digestion, which is considered as evidence that they are carried as internal exosomal cargo.

Here we showed that RIG-I, IFITM1, OAS2, Mx1 were upregulated in moDCs upon exposure to IK140508 exosomes (wt EBV) but not to RN exosomes (B95-8 EBV). However, IL-6 expression was upregulated by both types of EBV-modified exosomes. These results are consistent with a recent study which showed that exosomes from latent-EBV infected LCLs triggered antiviral immunity in dendritic cells through activation of cytosolic sensors [24]. We also showed that the different capacity of IFN-related gene induction between RN and IK140508 exosomes may due to the different copy numbers of EBER1 transferred into the moDCs.

The binding and internalization of PKH67-labeled EBV-modified exosomes into DonorI-HPV16 HFKs was observed by fluorescent imaging to be dose- and time-dependent. It is still unclear how exactly exosomes contact and enter recipient cells. It has been proposed that an acidic condition is critical for transfer of exosomes to recipient cells [36, 37]. A recent review suggested that extracellular vesicles (EVs) require a common viral entry pathway through receptor-ligand interaction with recipient cells for uptake [38]. Another study demonstrated that exosomes from type I and type III latency EBV-infected and EBV-uninfected B-cells could be internalized into uninfected epithelial cells in a similar fashion [39]. Our recent results confirm that EBV-modified exosomes are internalized by HFKs expressing HPV16 E6/E7 and the internal staining of PKH67 demonstrated the endocytic uptake. EBER1 was clearly detected in DonorI-HPV16 HFKs after incubation with EBV-modified exosomes for 24 hours and, upon PBS washing, remained stable at lower levels for 48 hours, as shown in Fig 4C. This demonstrated that EBER1 was transferred via exosomes into recipient cells and can persist for at least 2 days.

The effect of EBV-modified exosomes on DonorI-HPV16 cell proliferation was determined using the MTT assay. We did not observe any enhanced effect on cell proliferation. To our knowledge, we are the first group to demonstrate this. These HFKs may have different growth characteristics from epithelial cell lines used in previous studies. In addition, we also investigated the effects of EBV-modified exosomes on HPV oncogene expression but we did not observe the induction of these oncogenes either in DonorI-HPV16 or FK16A cells.

In addition to direct growth and oncogenic effects of EBV-modified exosomes, we investigated potential (pro) inflammatory effects of EBER1 transferred via exosomes into DonorI-HPV16 HFKs. We found that EBV-modified exosomes, both B95-8 and wt strain, did not induce the expression of IFN-related genes and IL-6 in DonorI-HPV16 or FK16A cells, but these genes were induced by 5 μg poly(I:C) which was used as a positive control. Tummers et al. recently demonstrated that high-risk HPV (hrHPV) upregulated the expression of IFRD1 to deregulate the K310 acetylation of NFκB/RelA in HFKs resulting in impairment of proinflammatory cytokine production [26]. EBERs-mediated IFN production is also induced via NFκB activation: thus it is possible that hrHPV deregulates this signaling pathway and suppresses IFN-related gene expression in HFK cells. To prove that E6/E7 expression in DonorI-HPV16 HFKs suppressed the induction of IFN response in these cells, HPV-negative primary HFKs were used in the experiments. Again, EBV-modified exosomes failed to induce IFN-related gene expression in primary HFKs. These results indicate that EBV-modified exosomes, which carry and transfer EBER1 in a physiologically natural condition, have no effect on the IFN production in HFKs. Considering the far lower levels of EBER1 molecules delivered into HFK cells via exosomes of RN or IK140508 LCLs, compared to transfection with ivt EBER1, our results indicate that the induction of IFN-related genes and IL-6 in HFK-hrHPV cells depends on quantity of EBER1 delivered. In addition, the different effects of EBV-modified exosomes in HFKs and moDCs demonstrate that induction of IFN-related genes and proinflammatory cytokines depends on types of recipient cells which have different characteristics, such as the ability to uptake antigen and viral detection by cytosolic sensors.

Rather than having a direct effect on (early-stage) HPV-transformed epithelial cells, our finding that EBER1, transferred via exosomes from latent-EBV infected B-cells, induces IFN-related gene expression in dendritic cells and can thus provide a link to inflammation-mediated tumorigenesis. It has been purposed that an inflammatory microenvironment is an essential component of tumors. Up to 20% of all tumors are associated with chronic inflammation [40] and inflammation by viral infection increases risk of cancer development [41]. DCs are one of the types of immune cells residing in the tumor microenvironment. These cells communicate with other immune cells or epithelial cells by direct contact or cytokine and chemokine production, which act in autocrine and paracrine manner. Deregulation of IFN-related gene expression in dendritic cells by EBER1 transferred via exosomes leads to unbalanced production of proinflammatory cytokines that activate transcriptional factors such as NFκB, STAT3 and AP-1 in premalignant cells resulting in stimulation of cell proliferation and survival. However, further studies are required to clarify the effects on HPV-infected keratinocytes of cytokines from dendritic cells which carry EBV products.

## Conclusions

EBV-modified exosomes could be internalized into HFKs expressing HPV16 E6/E7, thus transferring EBV small RNAs and miRNAs. Although lipid transfection of EBER1 triggered IFN-related gene expression, EBER1 transferred via exosomes had no effect on these HFKs. However, exosomes from wt EBV-infected LCLs induced IFN-related genes and IL-6 in moDCs. We propose that the induction of IFN-related genes and IL-6 by EBER1 depends on the quantity of EBER1 and the type of recipient cells. High levels of EBER1 in cervical cells may play a role in the inflammation-to-oncogenesis transition of HPV-associated cervical cancer development through the modulation of innate immune signals.

## Supporting Information

S1 Fig Expression of IFN-related genes and inflammatory cytokine gene in FK16A cells. EBV-modified exosomes were added to FK16A cells and incubated for 24 hours. The expression levels of IFN-related genes and inflammatory cytokine gene were determined by qRT-PCR. Relative expression level was corrected using the housekeeping gene GAPDH (2^-ddCp^). **; P < 0.01.
(TIF)

S2 Fig Expression of IFN-related gene and inflammatory cytokine gene in primary HFKs. EBV-modified exosomes were added to primary HFKs and incubated for 24 hours. The expression levels of IFN-related genes and inflammatory cytokine gene were determined by qRT-PCR. Relative expression level was corrected using the housekeeping gene GAPDH (2^-ddCp^). **; P < 0.01.
(TIF)

S1 Table Stem-loop primer sequences. (DOCX)

S2 Table qPCR primer and probe sequences and working concentrations. (DOCX)

S3 Table qPCR primer sequences and working concentrations for EBER1 quantification. (DOCX)

## Abbreviations

APC: antigen presenting cell
ANOVA: one-way analysis of variance
BPE: bovine pituitary extract
DC: dendritic cell
EBER: EBV-encoded RNA
EBV: Epstein-Barr virus
ECL: enhanced chemiluminescence
EGF: epidermal growth factor
EGFR: epidermal growth factor receptor
FBS: fetal bovine serum
GAPDH: glyceraldehyde 3-phosphate dehydrogenase
HFK: human foreskin keratinocytes
HPV: human papillomavirus
hrHPV: high-risk HPV
HSIL: high grade squamous intraepithelial lesion
IARC: international agency for research on cancer
IFITM: interferon-induced transmembrane protein
IFN: interferon
IFRD: interferon-related development regulator
IL-6: interleukin 6
IRF: interferon regulatory factor
ivt EBER1: *in vitro* transcribed EBER1
LCLs: lymphoblastoid cell lines
miRNAs: microRNAs
moDCs: monocyte-derived dendritic cells
MVBs: multivesicular bodies
Mx1: interferon-induced GTP-binding protein Mx1
OAS: 2′-5′ oligoadenylate synthetase
PAMP: pathogen-associated molecular pattern
PBMCs: peripheral blood mononuclear cells
PBS: phosphate buffer saline
PKR: protein kinase R
PRR: pathogen recognition receptor
qRT-PCR: quantitative reverse transcription polymerase chain reaction
RIG-1: retinoic acid-inducible gene 1
SCC: squamous cervical cell carcinoma
SDS-PAGE: sodium dodecyl sulfate-polyacrylamide gel electrophoresis
SEM: standard error of the mean
snRNP: small nuclear ribonucleic protein
TLR: Toll-like receptor
TNF: tumor necrosis factor
wt EBV: wild-type EBV
