# Supplementary figures and images for: High Levels of EBV-Encoded RNA 1 (EBER1) Trigger Interferon and Inflammation-Related Genes in Keratinocytes Expressing HPV16 E6/E7
Source: PLoS One. 2017 Jan 5;12(1):e0169290. doi: 10.1371/journal.pone.0169290 (PMC5215905; doi:10.1371/journal.pone.0169290)

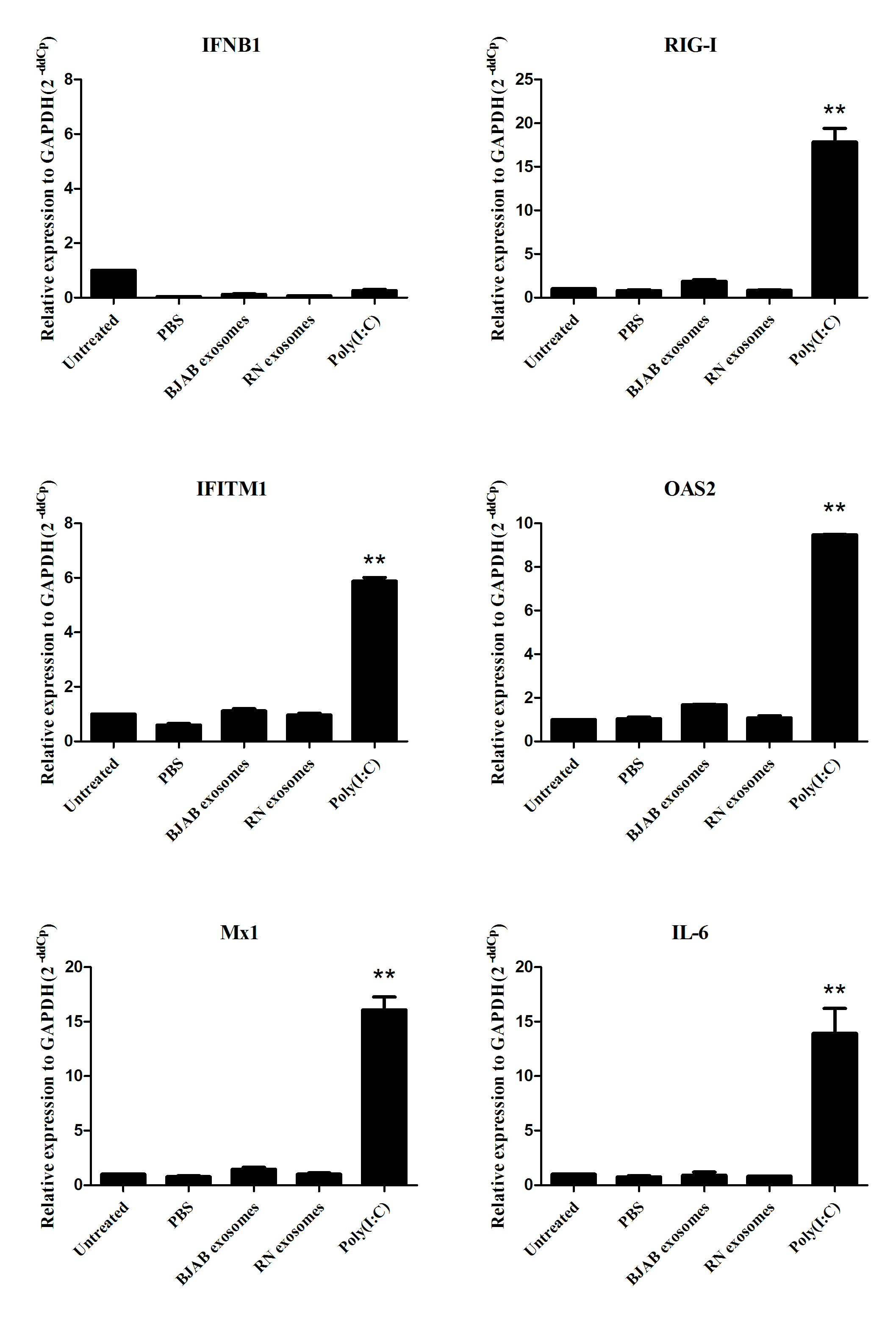

Supplement: S1 Fig — EBV-modified exosomes were added to FK16A cells and incubated for 24 hours. The expression levels of IFN-related genes and inflammatory cytokine gene were determined by qRT-PCR. Relative expression level was corrected using the housekeeping gene GAPDH (2-ddCp). **; P < 0.01. (TIF) [file pone.0169290.s001.tif]

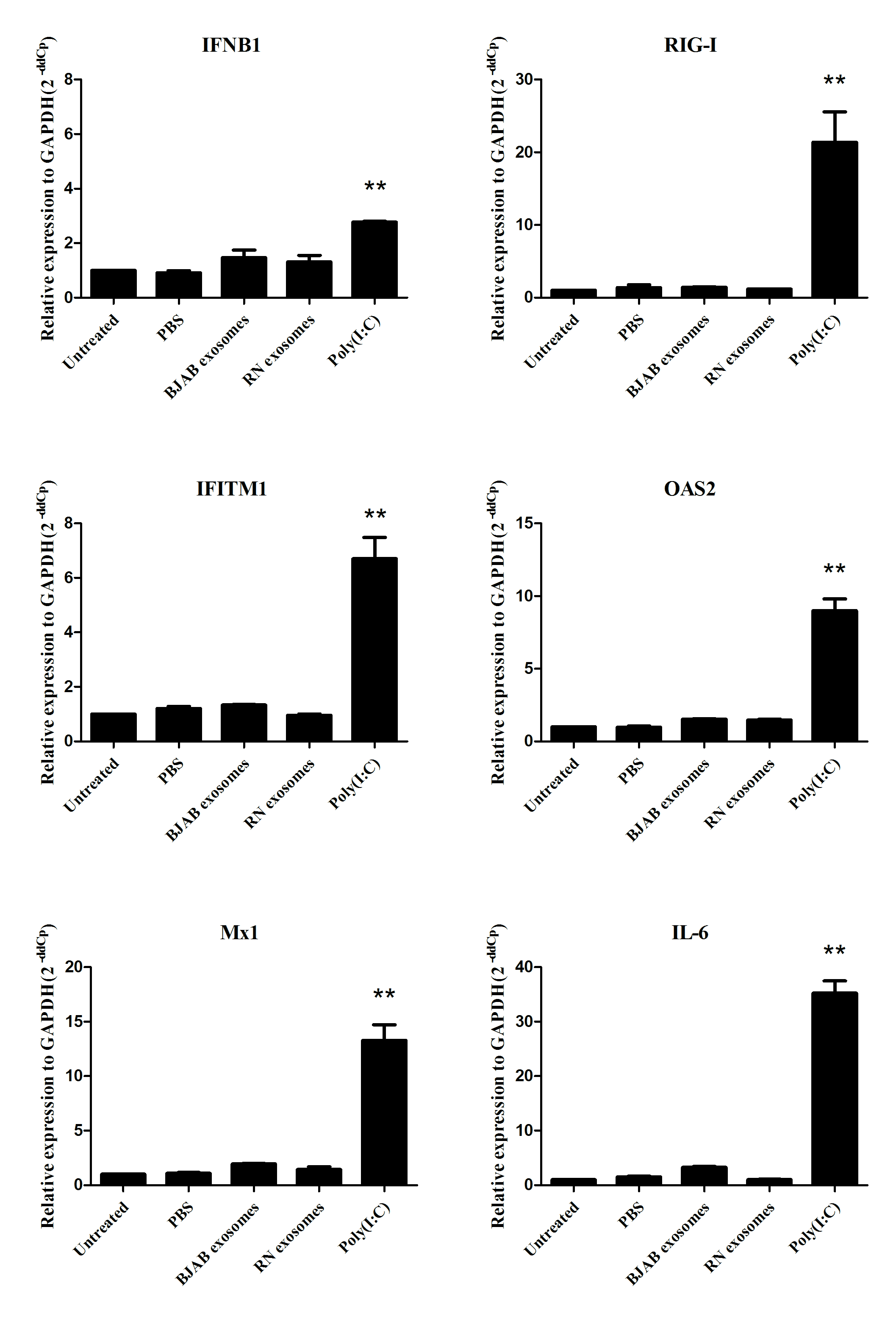

Supplement: S2 Fig — EBV-modified exosomes were added to primary HFKs and incubated for 24 hours. The expression levels of IFN-related genes and inflammatory cytokine gene were determined by qRT-PCR. Relative expression level was corrected using the housekeeping gene GAPDH (2-ddCp). **; P < 0.01. (TIF) [file pone.0169290.s002.tif]
