# Supplementary material for: High Levels of EBV-Encoded RNA 1 (EBER1) Trigger Interferon and Inflammation-Related Genes in Keratinocytes Expressing HPV16 E6/E7
Source: PLoS One. 2017 Jan 5;12(1):e0169290. doi: 10.1371/journal.pone.0169290 (PMC5215905; doi:10.1371/journal.pone.0169290)
Supplement: S1 Table — (DOCX) [file pone.0169290.s003.docx]

S1 Table. Stem-loop primer sequences

| **Primers** | **Sequences** (5’🡪3’) |
| --- | --- |
| RNY1 RNA (internal control) | CTCAACTGGTGTCGTGGAGTCGGCAATTCAGTTGAGAAAAGACTAG |
| EBER1 | CTCAACTGGTGTCGTGGAGTCGGCAATTCAGTTGAGAAAACATGCG |
| EBER2 | CTCAACTGGTGTCGTGGAGTCGGCAATTCAGTTGAGAAAAATAGCG |
