# Supplementary material for: High Levels of EBV-Encoded RNA 1 (EBER1) Trigger Interferon and Inflammation-Related Genes in Keratinocytes Expressing HPV16 E6/E7
Source: PLoS One. 2017 Jan 5;12(1):e0169290. doi: 10.1371/journal.pone.0169290 (PMC5215905; doi:10.1371/journal.pone.0169290)
Supplement: S2 Table — (DOCX) [file pone.0169290.s004.docx]

S2 Table. qPCR primer and probe sequences and working concentration

| Primers |  | Working concentration | Sequences (5’🡪3’) |
| --- | --- | --- | --- |
| RNY1 | Forward | 5.0 μM | GCTGGTCCGAAGGTAGTGAG |
|  | Reverse | 5.0 μM | ATTCAGTTGAGAAAAGACTAGT |
| EBER1 | Forward | 2.0 μM | AGGACCTACGCTGCCCTAGA |
|  | Reverse | 2.0 μM | ATTCAGTTGAGAAAACATGCGG |
| EBER2 | Forward | 2.0 μM | AGGACAGCCGTTGCCCTAGTGGTTTC |
|  | Reverse | 2.0 μM | ATTCAGTTGAGAAAAATAGCGG |
|  | Probe | 2.0 μM | FAM-TTCAGTTGAG TCTAAGGC-TAMRA |
| HPV16E6204s | Forward | 100 μM | AGCAACAGTTACTGCGACGTGAGG |
| HPV16E6502as | Reverse | 100 μM | CTGCAACAAGACATACATCGACCG |
| MP-GAPDH-F | Forward | 100 μM | GAGTCAACGGATTTGGTCGTAT |
| MP-GAPDH-R | Reverse | 100 μM | ATGGGTGGAATCATATTGGAAC |
| snRNP757F | Forward | 100 μM | TCCTCACCAACCTGCCAGA |
| snRNP828R | Reverse | 100 μM | TGAAGCCAGGGAACTGATTGA |
| snRNP778p | Probe | 5 μM | AGACCAACGAGCTCATGCTGTCCATG |
| MP-GAPDH-F | Forward | 5 μM | GAGTCAACGGATTTGGTCGTAT |
| MP-GAPDH-R | Reverse | 5 μM | ATGGGTGGAATCATATTGGAAC |
| IFITM1F | Forward | 5 μM | ATGTCGTCTGGTCCCTGTTC |
| IFITM1R | Reverse | 5 μM | GTCATCAGGATGCCCAGAAT |
| MP-IFNB1-F | Forward | 2 μM | CCTGAAGGCCAAGGAGTACA |
| MP-IFNB1-R | Reverse | 2 μM | AAGCAATTGTCCAGTCCCAG |
| MP-RIG1-F | Forward | 1 μM | ATATCCGGAAGACCCTGGAC |
| MP-RIG1-R | Reverse | 1 μM | GAGAAAAAGTGTGGCAGCCT |
| MP-IL6-F | Forward | 2 μM | AGTGAGGAACAAGCCAGAGC |
| MP-IL6-R | Reverse | 2 μM | CATTTGTGGTTGGGTCAGG |
| MP-OAS2-F | Forward | 2 μM | GGTGAACACCATCTGTGACG |
| MP-OAS2-R | Reverse | 2 μM | ACCATCGGAGTTGCCTCTTA |
| MP-MX1-F | Forward | 2 μM | GATTTTGGGGCTTTCCAGTC |
| MP-MX1-R | Reverse | 2 μM | GATGATCAAAGGGATGTGGC |
