# Supplementary material for: High Levels of EBV-Encoded RNA 1 (EBER1) Trigger Interferon and Inflammation-Related Genes in Keratinocytes Expressing HPV16 E6/E7
Source: PLoS One. 2017 Jan 5;12(1):e0169290. doi: 10.1371/journal.pone.0169290 (PMC5215905; doi:10.1371/journal.pone.0169290)
Supplement: S3 Table — (DOCX) [file pone.0169290.s005.docx]

S3 Table. qPCR primer sequences and working concentration for EBER1 quantification

| Primers |  | Working concentration | Sequences (5’🡪3’) |
| --- | --- | --- | --- |
| EBER2.2 forward primer | Forward | 10 µM | GAGGTTTTGCTAGGGAGGAGA |
| EBER1.1 reverse primer | Reverse | 10 µM | CGGACCACCAGCTGGTA |
